# The molecular basis of p21-activated kinase-associated neurodevelopmental disorders: From genotype to phenotype

**DOI:** 10.3389/fnins.2023.1123784

**Published:** 2023-03-02

**Authors:** Manon Dobrigna, Sandrine Poëa-Guyon, Véronique Rousseau, Aline Vincent, Annick Toutain, Jean-Vianney Barnier

**Affiliations:** ^1^Institut des Neurosciences Paris-Saclay, UMR 9197, CNRS, Université Paris-Saclay, Saclay, France; ^2^Department of Genetics, EA7450 BioTARGen, University Hospital of Caen, Caen, France; ^3^Department of Genetics, University Hospital of Tours, UMR 1253, iBrain, Université de Tours, INSERM, Tours, France

**Keywords:** intellectual disability, autism spectrum disorder (ASD), neurodevelopmental disorders, genotype/phenotype correlation, p21-activated kinase, mutations

## Abstract

Although the identification of numerous genes involved in neurodevelopmental disorders (NDDs) has reshaped our understanding of their etiology, there are still major obstacles in the way of developing therapeutic solutions for intellectual disability (ID) and other NDDs. These include extensive clinical and genetic heterogeneity, rarity of recurrent pathogenic variants, and comorbidity with other psychiatric traits. Moreover, a large intragenic mutational landscape is at play in some NDDs, leading to a broad range of clinical symptoms. Such diversity of symptoms is due to the different effects DNA variations have on protein functions and their impacts on downstream biological processes. The type of functional alterations, such as loss or gain of function, and interference with signaling pathways, has yet to be correlated with clinical symptoms for most genes. This review aims at discussing our current understanding of how the molecular changes of group I *p21-activated kinases* (*PAK1*, *2* and *3*), which are essential actors of brain development and function; contribute to a broad clinical spectrum of NDDs. Identifying differences in *PAK* structure, regulation and spatio-temporal expression may help understanding the specific functions of each group I *PAK*. Deciphering how each variation type affects these parameters will help uncover the mechanisms underlying mutation pathogenicity. This is a prerequisite for the development of personalized therapeutic approaches.

## Introduction

The 5th edition of the Diagnostic and Statistical Manual of mental disorders (Crocq and Guelfi, 2015) defines neurodevelopmental disorders (NDDs) as a broad class of brain diseases characterized by a spectrum of early clinical manifestations with developmental delay and cognitive/social impairments representing the most recurrent phenotypes. A non-exhaustive list of NDDs includes intellectual disability (ID), Autism Spectrum Disorders (ASD), Attention-Deficit/Hyperactivity Disorder (ADHD), developmental epilepsies and motor disorders such as cerebral palsy. Schizophrenia (SCZ) and Bipolar disorders (BP) could also be considered as NDDs (Crocq and Guelfi, 2015; Michetti et al., 2022). These psychiatric and neurological disorders participate in a network of neuropsychiatric diseases that share etiology and clinical commonalities related to their complex nosology (i.e., classification of diseases) (Vissers et al., 2016; Owen and O’Donovan, 2017; Glasson et al., 2020; Morris-Rosendahl and Crocq, 2020; Leblond et al., 2021; Vilela et al., 2022). Next-generation sequencing (NGS) has helped identify pathogenic variants in many genes for a large proportion of patients presenting ID and highlights the genetic complexity in NDDs (de Ligt et al., 2012). For instance, more than 1,000 genes have been associated with ID risk and more than 400 with ASD (Willsey et al., 2017; OMIM, 2022). The large number of genes involved makes the identification of pathogenic variants by clinicians and geneticists extremely complex (Piton et al., 2013; Wright et al., 2015; Chiurazzi and Pirozzi, 2016). Hence, distinguishing deleterious variants from neutral polymorphisms is still a challenge for many single nucleotide variations (SNVs), even if they are located in coding sequences. Moreover, different mutations in the same gene can lead to a broad spectrum of clinical symptoms, which makes the etiological characterization of NDDs difficult. An ongoing challenge is to understand how various genetic changes can cause different NDDs. The many approaches developed based on robust algorithms and systematically updated databases open the possibility of elaborating frameworks of analysis for the relationship between genetics and clinics (Gussow et al., 2016; Geisheker et al., 2017; Sanders et al., 2019).

**FIGURE 1:**
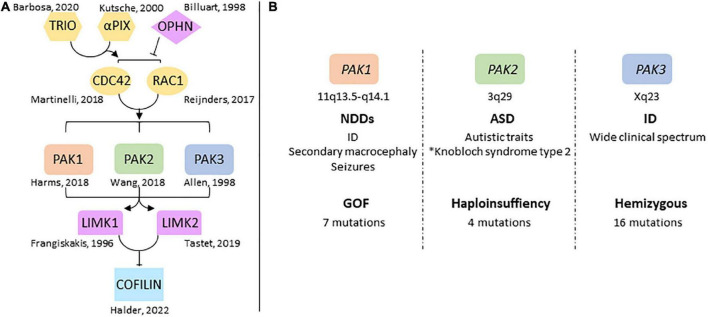
Overview of group 1-PAKs in NDDs **(A)** genes of the RAC1/CDC42 pathway are involved in NDDs. The identification of pathogenic variations in several genes highlights the central role the dysregulation of this pathway plays in NDDs, notably via its control of actin cytoskeleton dynamics through cofilin. **(B)** Chromosomal location of PAK genes and conditions generally associated with PAK variations. PAKs are associated with very different mutational and clinical landscapes. *Antonarakis et al. (2021).

The genes involved in NDDs regulate central signaling pathways that often share a limited number of functions such as chromatin regulation, proliferation and differentiation of neural stem cells, and synaptic plasticity (Ernst, 2016; Forrest et al., 2018; Moretto et al., 2018; Liaci et al., 2021). This convergence of signaling pathways in NDDs can help identify common therapeutic targets. Thus, uncovering the way signaling pathways are disrupted remains a priority to better understand the pathology and define new therapeutic approaches. The RAC1/CDC42 pathway is central to brain development and function since it regulates actin cytoskeleton dynamics, which is essential to cell migration, synaptic plasticity, axon guidance and neurogenesis (Nadif Kasri and Van Aelst, 2008; van Bokhoven, 2011; Penzes and Cahill, 2012; Gentile et al., 2022). The switch between cytosolic, GDP-loaded inactive-GTPases and membrane-bound, GTP-loaded active forms usually regulates this pathway. Two large families control these states: the Guanine Exchange Factor (GEF) and the GTPase-Activating Proteins (GAP). Variations in *RAC1*, *CDC42* as well as those in several GEF such as the *PIX* guanine exchange factors, and in several GAP such as *Oligophrenin*, and other genes connected to this RHO GTPase pathway are responsible for NDDs, epilepsy and are often associated with brain structural anomalies (Figure 1A; Frangiskakis et al., 1996; Billuart et al., 1998; Kutsche et al., 2000; Reijnders et al., 2017; Martinelli et al., 2018; Tastet et al., 2019; Barbosa et al., 2020; Halder et al., 2022). Genetic data also helped identify pathogenic variations in *Lim Kinases* and *cofilin* involved in neuropsychiatric conditions, confirming the role of the RAC/CDC42/PAKs/LimK/Cofilin pathway in NDDs (Frangiskakis et al., 1996; Tastet et al., 2019; Halder et al., 2022). Cofilin activity is regulated by LIM kinase-dependent phosphorylation, Slingshot-dependent dephosphorylation, and other interactions with protein partners. Cofilin controls dendritic spine dynamics and synaptic expression of glutamate receptors in the post-synaptic compartment, as well as vesicle exocytosis and neurotransmitter release in the presynaptic compartment (Ben Zablah et al., 2020).

p21-activated kinases (PAKs) mediate several functions downstream of RAC1 and CDC42. They regulate actin cytoskeleton dynamics via Lim kinase activation and phosphorylation-dependent cofilin inhibition (Bokoch, 2003; Boda et al., 2010; Ben Zablah et al., 2020; Zhang K. et al., 2022). PAKs are divided in two groups according to their sequence identity, their structural properties, and regulatory mechanisms (Eswaran et al., 2008). Group I *PAKs* includes *PAK1*, *2* and *3*; group II includes *PAK4, 5* and *6* which have not been associated with NDDs yet (Jaffer and Chernoff, 2002; Bokoch, 2003). Several reviews describe the biological functions of PAKs in cell signaling and neuronal pathophysiology (Bokoch, 2003; Kreis and Barnier, 2009; Koth et al., 2014; Kumar and Li, 2016; Civiero and Greggio, 2018; Wang and Guo, 2022; Zhang K. et al., 2022). Functions of group I PAKs are similar in a broad way because of their high sequence identity, their common modes of regulation as well as the substrates they share. Recent data from human genetics highlight their implication in NDDs. Pathogenic variations of *PAK1*, *PAK2* and *PAK3* are responsible for ID and/or psychiatric disorders, and are often associated with brain anomalies (Allen et al., 1998; Harms et al., 2018; Wang et al., 2018). However, the clinical spectrum associated with mutations in each *PAK* genes is different (Figure 1B). This review aims to provide an overview of the different clinical cases associated with *PAKs* thus far, to reflect on the effects of mutations on PAK functions, and how *PAK* expression may be involved in the symptomatology of *PAK*-associated disorders.

## History of case reports and clinical data

### *PAK3*: From non-syndromic XLID to a complex clinical pattern

**FIGURE 2:**
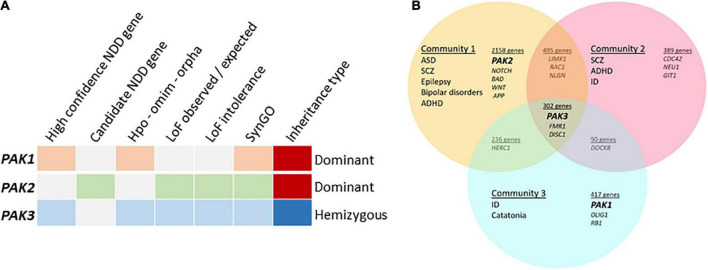
Meta-analyses of group I PAK-associated NDDs **(A)** PAKs in different databases, categories highlighted when PAK1 (orange), PAK2 (green) or PAK3 (blue) are detected. The high confidence NDD gene category was created by GeneTrek (Leblond et al., 2021) using data-mining from SFARI, SPARK, SyslD, DDG2P, and DBD databases. The candidate NDD gene category includes candidate risk genes from the same databases (except SPARK). HPO-OMIM-Orpha: other databases. Loss of function (LOF) observed or expected: LOF can be expected from genes in this category. LOF intolerance corresponds to genes for which LOF leads to pathogenic effects. Genes from SYNGO database encode synaptic proteins. **(B)** *PAK* genes are associated with different community of diseases identified by Vilela et al. (2022).

**FIGURE 3:**
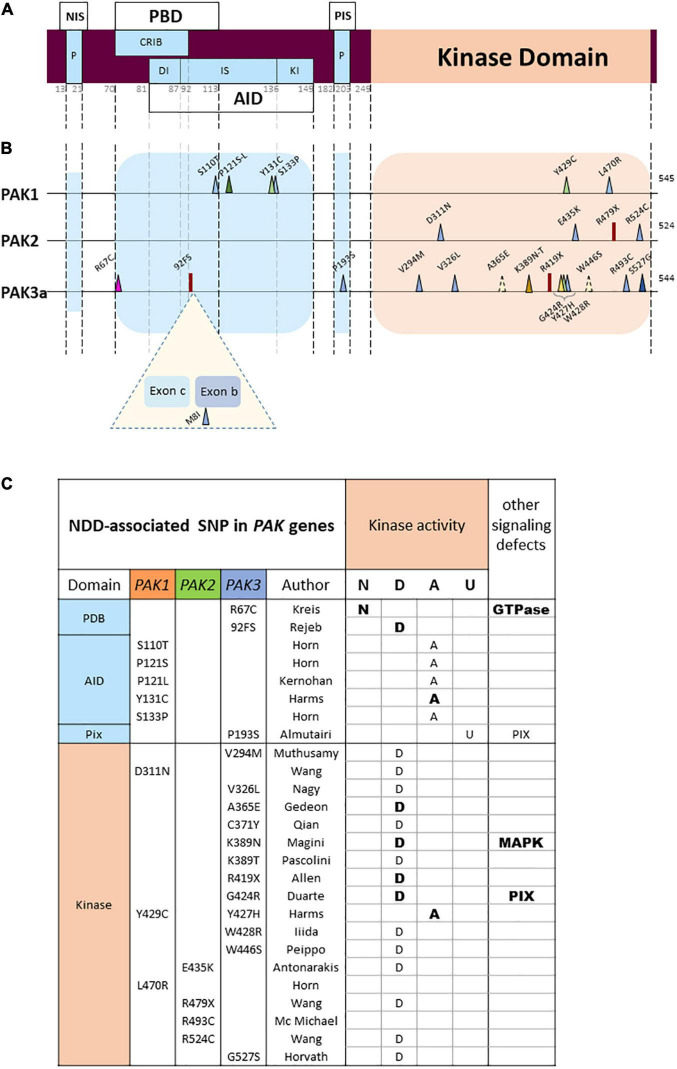
PAK general structure and localization of variants **(A)** Domain structure of PAKs. NIS, Nck interacting sequences; P, praline-rich sequences; PBD, p21 binding domain; AID, auto-inhibitory domain; DI, Dimerization segment; IS, Inhibitory switch domain; Kl, kinase inhibitory segment; PIS, PIX interacting sequences. Numbering of the general structure is based on PAK 1. **(B)** Location of missense and LGD (likely gene disruptive) variations. Darker colors represent reccurrences. Both PAK3 alternative exons are represented (Exon b: 15 amino-acids; exon c: 21 amino-acids). **(C)** Validated or hypothetical functional alterations of *PAK* variants. N: normal kinase activity; D: defective; A: activated; U: unknown. Bold: experimentally validated; normal: hypothetical.

We briefly describe here the history of the identification of *PAK* genetic variants and the implementation of concepts that have accompanied these discoveries over the past 25 years (Table 1). The autosomal location of the two *PAK1* and *PAK2* genes explains the delayed timing of the discovery of their genetic involvement in NDDs, compared to the X-linked *PAK3* gene. The first evidence of a genetic factor in ID comes from studies of its transmission to men in large families and the analysis of the segregation of chromosomal markers. Because of the male-to-female ratio of patients affected with ID, the X chromosome has been linked to this pathology early on (Lehrke, 1972; Chelly and Mandel, 2001; Gécz et al., 2009; Lubs et al., 2012). Variant identification was carried out by analyzing karyotypes and segregation of markers, thus requiring cross-referencing of data from several patients. Then, the advent of NGS has made it possible to identify variations in the DNA of isolated patients. The first pathogenic mutation in the *PAK3* gene was discovered in a family (MRX30) described in 1996. The region involved was mapped between Xq21.3 and Xq24 (Donnelly et al., 1996) and the variant was later identified as p.(Arg419*), and corresponds to a nonsense mutation in the sequence encoding the kinase domain of the *PAK3* gene (Allen et al., 1998). The second variant identified, p.(Arg67Cys), was the first missense mutation found within the *PAK3* gene (des Portes et al., 1997; Bienvenu et al., 2000). The MRX47 family bearing this mutation was previously described in 1997 (des Portes et al., 1997). This mutation localized in the regulatory domain modifies PAK3 affinity for GTPases (Kreis et al., 2007). The p.(Ala365Glu) substitution within a highly conserved region of the kinase domain was detected in 19 males in a family spread over five generations. Patients bearing this *PAK3* variant were diagnosed with mild non-syndromic X-linked ID with a few individuals also presenting neuropsychiatric problems such as aggressiveness, antisocial behavior, psychosis, depression or epilepsy (Gedeon et al., 2003). In 2007, a p.(Trp446Ser) mutation, located in the kinase domain and affecting 12 patients from a four-generation family was published (Peippo et al., 2007). It was the first description of learning difficulties in four carrier females that displayed borderline to mild ID (Peippo et al., 2007). Two males had drooling, otherwise all four males presented inarticulate speech, short attention span, anxiety, restlessness and aggressiveness and one of them had a small head. These data indicate that *PAK3* variations can be associated with psychiatric traits and brain anomalies, thus being responsible for a more complex clinical description than non-syndromic ID (Figure 2). Indeed, microcephaly is now often associated with *PAK3* variations. Notably, in 2008, two brothers bearing the first frameshift variant due to a four-bases insertion at the end of the sixth exon p.(Gly92Valfs*35), located after the second coding exon were described with ID, aggressiveness and microcephaly (Figure 3; Rejeb et al., 2008). This clinical report, combined with the previous literature, prompted these authors to suggest that *PAK3*-associated disorders should be considered as a distinguishable ID syndrome. In 2014, the p.(Lys389Asn) mutation associated with severe ID was described in two boys that displayed microcephaly, corpus callosum agenesis (CCA) and cerebellar hypoplasia and one fetus that displayed CCA and septum pellucidum agenesis (Magini et al., 2014). The two patients later died from epileptic seizures at age 6 and 8. The *in vitro* and *in vivo* analysis of the functional consequences of this variant indicated a large effect on cell signaling and on craniofacial structures. A less severe case was described soon after, the p.(Arg493Cys), responsible for borderline ID, epilepsy and hemiplegic cerebral palsy (McMichael et al., 2015). During the same period, the p.(Val294Met) was identified in patients displaying moderate ID, ADHD and microcephaly (Muthusamy et al., 2017). Soon after, a missense mutation, p.(Tyr427His), described in monozygotic twins, was associated with macrocephaly in addition to developmental delay, ID and autistic features, such as sensory processing issues (Hertecant et al., 2017). It was the first time macrocephaly was associated with *PAK3* mutations. More recently, a patient bearing a p.(Ser527Gly) mutation, located in the kinase domain, was described with severe ID, epilepsy, and self-injurious behavior (Horvath et al., 2018). Since 2018, almost only severe ID was described, such as the p.(Val326Leu) variation in a boy, the p.(Trp428Arg) variation in two siblings or the p.(Gly424Arg) variation in a familial case (Iida et al., 2019; Duarte et al., 2020; Nagy et al., 2020). The latter case displayed global developmental delay, stereotypies, and was later diagnosed with secondary microcephaly in addition to ID and corpus callosum dysgenesis. The same pathogenic variant was found retrospectively in the fetus from a previous pregnancy terminated after detection of CCA and hydrocephaly (Duarte et al., 2020). It was not until 2021 that female cases were reported as probands. Indeed, some pathogenic variations are now described in girls. The first variation responsible for such a case is the p.(Lys389Thr), which was also the second described substitution affecting the Lys389 residue. The patient bearing this mutation displayed ID, secondary microcephaly and ADHD (Pascolini et al., 2021). The published p.(Pro229Ser) variation indexed in *PAK3cb*, which corresponds to a p.(Pro193Ser) variation in the *PAK3a* isoform (Figure 3B), is associated with ID, microcephaly and combined immunodeficiency syndrome in the proband girl (Almutairi et al., 2021). To summarize, the clinical picture associated with *PAK3* mutations was initially characterized as non-syndromic ID but the identification of additional *PAK3* mutations has unveiled the comorbidity of ID with many psychiatric traits as well as morphological abnormalities of the brain. *PAK3* mutations can lead to ID of varying severity and may be associated with autistic traits, ADHD, paranoid psychosis, aggressiveness, self-harm, and other neurological signs such as epilepsy and motor disorders.

**TABLE 1:** Historical discoveries of *PAK* variations in NDDs.

Mutation	*PAK1*	*PAK2*	*PAK3*	References
p.(Arg419*)			1st mutation, mild ID, ADHD, microcephaly	Allen et al., 1998
p.(Arg67Cys)			1st missense mutation, moderate to severe ID	Bienvenu et al., 2000
p.(Ala365Glu)			Mild ID, variable psychiatric presentations	Gedeon et al., 2003
p.(Trp446Ser)			Mild to moderate ID, ADHD, agressiveness	Peippo et al., 2007
p.(Gly92Valfs*35)			1st Frame-shift, mild to moderate ID, agressiveness, microcephaly	Rejeb et al., 2008
p.(Lys389Asn)			Severe ID, ASD, epilepsy, microcephaly, CCA, cerebellar hypoplasia	Magini et al., 2014
p.(Arg493Cys)			Borderline ID, epilepsy, hemiplegic cerebral palsy	McMichael et al., 2015
Xq23 deletion, PAK3 exons (4-15)del			Mild ID, developmental delay, ASD	Cartwright et al., 2017
p.(Val294Met)			Moderate ID, ADHD, microcephaly	Muthusamy et al., 2017
p.(Tyr427His)			Severe ID, ASD, macrocephaly	Hertecant et al., 2017
p.(Ser527Gly)			Moderate ID, ASD, seizures, ventriculomegaly	Horvath et al., 2018
p.(Asp311Asn) p.(Arg479*) p.(Arg524Cys)		Haploinsufficiency, ASD		Wang et al., 2018
p.(Tyr131Cys) p.(Tyr429Cys)	2 dominantly-acting Gain Of Function (GOF), ID, epilepsy, macrocephaly			Harms et al., 2018
p.(Val326Leu)			Mild to moderate ID, ASD, microcephaly	Nagy et al., 2020
p.(Trp428Arg)			Severe ID, ASD, microcephaly, CCA	Iida et al., 2019
p.(Ser110Thr) p.(Pro121Ser) p.(Ser133Pro) p.(Leu470Arg)	Moderate to severe ID, seizures, macrocephaly, ventriculomegaly			Horn et al., 2019
p.(Pro121Leu)	ID, ASD, epilepsy, macrocephaly			Kernohan et al., 2019
p.(Gly424Arg)			Severe ID, ASD, microcephaly, CCA	Duarte et al., 2020
p.(Lys389Thr)			1st female case, ADHD, microcephaly	Pascolini et al., 2021
p.(Pro193Ser)			Female patient, mild ID, ASD, Microcephaly	Almutairi et al., 2021
p.(Glu435Lys)		Knobloch type 2 syndrome		Antonarakis et al., 2021

Historical contexts of variant discoveries in *PAK1* and *PAK2* are very different from that of *PAK3*: more recent, concerning fewer patients, and associated with more homogeneous clinical spectra. Peptide numbering corresponds to Human *PAKs*, and the Human *PAK3a* splice variant in the case of *PAK3* variations.

### *PAK1* in developmental delay and macrocephaly

The identification of deleterious variations in the two other *PAK* genes is recent. The first mutation in the *PAK1* gene, p.(Arg500His), was reported in a meta-analysis of more than 2,000 patients with ID, but *PAK1* was not considered as a gene responsible for ID at that time (Lelieveld et al., 2016). In 2018, two p.(Tyr131Cys) and a p.(Tyr429Cys) mutations were identified in two unrelated patients displaying macrocephaly, ID, seizures, speech impairment and attention deficits (Harms et al., 2018). In 2019, other *PAK1* variants including p.(Ser133Pro), p.(Ser110Thr), p.(Pro121Ser) and p.(Leu470Arg) were reported in subjects with ID, macrocephaly and seizures in three boys and one girl, respectively, (Horn et al., 2019; Ohori et al., 2020). They are localized either in the regulatory domain or in the kinase domain such that their translated variants are predicted to suppress the auto-inhibitory mechanism, thereby directly activating or indirectly leading to PAK1 activation. In 2019, a second occurrence affecting the Pro121 residue was reported. This p.(Pro121Leu) mutation is responsible for an autosomal dominant disorder with severe regressive ASD, ID and epilepsy (Kernohan et al., 2019).

### *PAK2* variants are associated with ASD

The *PAK2* gene is localized at the end of the long arm of chromosome 3 and belongs to the 3q29 locus that is relatively frequently rearranged in patients with NDDs. The 3q29 duplication syndrome is associated with ID, microcephaly, cerebral palsy, and epilepsy. The minimal duplicated interval involves 22 annotated genes, including *PAK2* and *DLG1* (Lisi et al., 2008; Fernández-Jaén et al., 2014; Wen et al., 2022). Likewise, deletion of the 3q29 locus is associated with ASD (Sanders et al., 2015). Clinical studies reported ASD in patients bearing *PAK2* missense mutations or with *PAK2* deletions, causing *PAK2* haploinsufficiency (Wang et al., 2018). One missense p.(Glu435Lys) variation was recently identified by exome sequencing in two siblings displaying a Knobloch-like syndrome designated as KNO2 with ID, autistic behavior, retinal degeneration, and encephalocele (Antonarakis et al., 2021).

Despite the descriptions above, non-cognitive recurring symptoms have also been identified for several patients bearing *PAK1* or *PAK3* mutations. Some traits such as general hypotonia, drooling, nystagmus and strabismus, speech difficulties, and tremor, that may be due to neurological and/or neuromuscular defects, are described for patients bearing *PAK1* or *PAK3* variations. There are also some non-neurological symptoms associated with *PAK* variants. Interestingly, skin anomalies, such as café-au-lait spots, were described for the majority of patients bearing *PAK1* mutations and associated with some *PAK3* variations (Magini et al., 2014; Hertecant et al., 2017; Harms et al., 2018; Pascolini et al., 2021). These anomalies may be due to defects in the activation of the Ras-MAP kinase pathway, leading to RASopathy (Magini et al., 2014). Facial dysmorphic features are also commonly associated with *PAK1* and *PAK3* variations (deeply set eyes, large ears, hair alopecia, bushy eyebrows, nose shape, palate, etc.). A gross analysis suggests that the more severe the cognitive defects, the more non-neurological symptoms are present. This is probably due to the more damaging PAK defects in these cases. It should be noted that there is still no thorough clinical description of patients bearing *PAK2* variants, except for the KNO2 syndrome.

### Clinical overview of *PAK* variants

The description above indicates that variations are associated with different conditions depending on each *PAK* gene: *PAK1* pathogenic variants are associated with developmental delay, ID, ASD and macrocephaly, *PAK2* variants with ASD only and *PAK3* variants with ID, often along with ASD, ADHD, epilepsy, and, in numerous cases, microcephaly. The pathophysiological landscape associated with each *PAK* may also be observed from genetic database mining or from meta-analysis data in web sites such as GeneTrek, which surveys the association of human genes with NDDs (Figure 2A; Leblond et al., 2021). This could also be illustrated by the analysis of disease communities based on comorbidities and clinical characteristics in a disease network approach to explore the shared genetics between ASD, ID, ADHD, SCZ, BP, and epilepsy (Figure 2B; Vilela et al., 2022). As expected, the three *PAK* genes segregate with different disease communities. *PAK1* is found within the community mostly defined by ID, *PAK2* is found in the community mostly characterized by ASD, though the comorbidities associated with ASD make this community highly heterogeneous. Indeed, around 70% of individuals with ASD display at least one co-occurring NDD or form of epilepsy (Rosen et al., 2018). Furthermore, even though *PAK2* variations are mostly associated with ASD, the number of cases described at present is not sufficient to get a precise idea of the extent of clinical consequences. For example, the *PAK2* mutation associated with KNO2 syndrome (Antonarakis et al., 2021) already differs from previous descriptions of *PAK2* clinical cases that were exclusively associated with ASD. For *PAK3*, there is a large clinical variability so that *PAK3* is found in all three communities (Figure 2B). However, the exhaustive list of clinical traits associated with *PAK3* pathogenic variations is difficult to establish because of heterogeneous clinical descriptions of the patients, such as the relative importance given to certain traits compared to others or the context of medical practices. The absence of mutation hot spots precludes the description of a precise *PAK3*-associated syndrome and prevents ruling out effects other mutations or the environment might have on *PAK3*-associated NDDs (Lelieveld et al., 2016).

The meta-analyses previously mentioned (Leblond et al., 2021; Vilela et al., 2022) highlight the complex relationship between *PAK* variations and the extent of neuropsychiatric symptoms. Several reasons might explain the differences observed in the clinical symptoms associated with each *PAK*: the paucity of described variations, especially for *PAK1* (7) and *PAK2* (4), and the different functions of each *PAK* isoform. Furthermore, the scarcity of biochemical characterization of PAK variants limits our understanding of these differences. To summarize, the study of *PAK*-associated disorders is currently incomplete due to several hindrances. Yet, the improvement of variant detection methods, the homogenization of clinical analyses and the collaborative approaches between clinicians and researchers will support a more thorough approach.

## *PAK* genetic context governs the expression of patient conditions

An important difference between *PAKs* is that the variations in *PAK1* and *PAK2* genes are transmitted according to an autosomal and dominant inheritance pattern, while those in *PAK3* are X-linked and hemizygous. Both *PAK1* and *PAK2* are autosomal genes, respectively, located on chromosome 11 and 3. Pathogenic variations in *PAK1* are monoallelic *de novo* mutations displaying dominant functional effects (Harms et al., 2018; Horn et al., 2019; Kernohan et al., 2019). The missense *PAK2* variations are *de novo* or inherited (Zhang K. et al., 2022). The newly described *PAK2* mutation, responsible for the KNO2 syndrome, is monoallelic and detected in both siblings, indicating a likely germ-line mosaicism in one of the parents (Antonarakis et al., 2021). Whether this variant is responsible for haploinsufficiency is still unknown. For *PAK3*, most variants are inherited and only two of them, p.(Lys389Thr) and p.(Ser527Gly) occurred *de novo* (Horvath et al., 2018; Pascolini et al., 2021).

Variants in genes localized on the X chromosome form a special class because of the unique mode of allele expression: in males, the mutated allele is expressed in all cells whereas there is a mosaic expression of only one of the two alleles in female cells. In this instance the genetic approaches for patient cohorts with X-linked disorders is particular (Piton et al., 2013; Leitão et al., 2022). Until 2014, *PAK3* mutations were identified in large families over several generations and were found to induce less severe symptoms than in most recent cases, whom mutations are responsible for severe ID associated with other psychiatric traits. This change in case reports is probably due to a bias induced by the evolution of diagnostic methods, such as NGS, and to the fact that PAK3 is now more investigated because it is recognized as a gene involved in NDDs. The women bearing *PAK3* mutations were either unaffected female carriers or mothers presenting borderline to moderate ID (Peippo et al., 2007; Rejeb et al., 2008). Although skewed X-inactivation has been observed in several women, this could not be correlated with clinical characteristics. Unfortunately, there are no further data on more recently identified female probands (Almutairi et al., 2021; Pascolini et al., 2021). Despite the difference between men and women in *PAK*-associated disease transmission, no evidence suggests that differences in PAK functions exist between the two sexes.

Another observation is that each *PAK* displays a unique mutational pattern (Figure 3B). The landscape of genetic abnormalities of the three *PAK* genes is also particular insofar as current published data are mainly made up of missense mutations. For the three genes combined, there are twenty-three missense mutations, two nonsense mutations and one splice site mutation inducing a frameshift, as well as one multi-exonic deletion (Cartwright et al., 2017). There are only few recurrences. One double occurrence is on the *PAK1* proline 121 residue (Horn et al., 2019; Kernohan et al., 2019). For *PAK3*, two identical mutations p.(Ser527Gly) have been published as well as two variants affecting the same lysine 389 residue (Magini et al., 2014; Tzschach et al., 2015; Horvath et al., 2018; Pascolini et al., 2021). Interestingly, two mutations affecting homologous residues in the PAK1 and PAK3 paralogs, the p.(Tyr429Cys) and p.(Tyr427His), respectively, lead to similar clinical phenotypes associating ID, ASD and macrocephaly (Hertecant et al., 2017; Harms et al., 2018). Of note, the low number of described cases may explain the low number of recurrences and the absence of mutation hotspots.

## *PAK* mutations affect kinase functions, mechanisms of regulation and partner interactions

Nonsense mutations, monogenic deletion, and splice site mutations inducing a frameshift are qualified as Likely Gene Disrupting variations (LGD) that are responsible for loss of function or loss of protein (Figure 3B). These mutations could lead to truncated forms lacking kinase activity and exerting dominant-negative effects or signaling interference, but this possibility has not been thoroughly explored yet. However, mRNAs bearing premature termination codon can be degraded by the translation control system, named Nonsense-Mediated mRNA decay (Supek et al., 2021). This would lead to the total absence of defective proteins, responsible for a complete loss of function. *PAK3* LGD mutations are responsible for relatively mild to moderate severity of the disease. The *PAK2* gene is considered intolerant to loss-of-function (Figure 2A), and the *PAK2* nonsense mutation is also associated with haploinsufficiency. Furthermore, heterozygous LGD mutations in ASD-related genes are frequently predicted to cause haploinsufficiency (Parenti et al., 2020).

Other mutations are missense mutations and their variations could be associated with Loss Of Function (LOF) or Gain Of Function (GOF). In the case of kinases, these denominations correspond to complete loss of kinase activity or high constitutive kinase activity, respectively. This classification as LOF and GOF may be used to qualify the functional defects of the PAK1, PAK2 and some PAK3 variants. However, it does not cover the full range of functional alterations observed for other PAK3 variants. Indeed, PAKs are multifunctional proteins, with a kinase activity but also scaffolding functions, thus leading to kinase-dependent and kinase-independent roles. The best described of these kinase-independent functions is the recruitment of PIXs, which makes it possible to assemble a GEF/GTPase/effector complex, thus ensuring specificity and efficiency of the GEF/GTPase/PAK module (Obermeier et al., 1998). In other words, interactions with certain partners such as α/βPIX, Nck1/2 and Grb2 promote the formation of signaling complexes associated with membrane recruitments, and mutations in these interaction domains could significantly affect PAK signaling. Finally, pathogenic variations have only been identified in the coding sequences of group *I PAK*s. Given the importance of non-coding region variations in the etiology of NDDs, it would be advisable to look more closely for these kinds of changes (Takata, 2019).

PAK1 to 3 share an overall structure composed of a carboxy-terminal catalytic domain and an amino-terminal regulatory region (Figure 3A; Bokoch, 2003). Notwithstanding, PAK amino-acid sequences diverge in specific areas and those primary sequence differences, that are conserved among vertebrates, may support specific functions. The regulatory region consists of two overlapping domains, one is the p21-binding domain (PBD), and the other one is an auto-inhibitory domain (AID). The kinase domain comprises two lobes: the small lobe in N-ter is essentially composed of β-sheets while the larger lobe in C-ter is mainly constituted of α-helixes, in which several residues can interact with the auto-inhibitory domain (Lei et al., 2000; Parrini et al., 2002). In resting conditions, when PAKs are inactive, they form dimer comprised of two molecules in an asymmetric, antiparallel, manner, one monomer adopting an active conformation, and the other one an inactive (Wang et al., 2011). This *trans-inhibited* dimer is activated by several mechanisms that modify its conformation, induce a multi-step dissociation, and trigger several events of *cis*- and *trans-phosphorylation*. It is, however, possible that there was a bias in some crystallographic analyses, since the kinase-dead variant of PAK1 retains a very low, but sufficient, residual activity that allows its autophosphorylation (Wang et al., 2011). The crystallization carried out in the presence of phosphatases suppress this residual phosphorylation and generates inactive proteins in a monomeric conformation. Furthermore, the first step in the activation process corresponds to the transient formation of dimers (Sorrell et al., 2019).

Almost all pathogenic mutations currently identified are located in two domains corresponding to the kinase domain and the PBD/AID regulatory domain (Figure 3B), a fact that is probably due to the strong structural constraint observed in the catalytic and regulatory domains of kinases. In contrast, non-pathogenic single nucleotide polymorphisms (SNPs) extracted from databases are mainly distributed in sequences coding for non-functional, more flexible, zones. Note also the seemingly total penetrance of *PAK1* and *PAK2* mutations, as well as *PAK3* mutations in boys.

In addition to the slight structural differences between the three group I PAKs, the *PAK3* gene also shows specificity as it can encode four splice variants (Rousseau et al., 2003; Kreis et al., 2007). PAK3a is devoid of any insertion between the position 92 and 93 of the amino acid sequence. PAK3b contains an insertion of 15 amino acids at this position. PAK3c displays an insertion of 21 amino acids, located between the coding exon 2 and exon b. Finally, PAK3cb (also called PAK3d) exhibits both b and c alternative exons (Figure 3B). To date, a gross characterization of the expression of PAK3 splice isoforms has been conducted using semi quantitative RT-PCR, since the splice variant-specific antibodies available at present cannot work for immunofluorescent detection. All spliced isoforms have been detected in the mouse adult brain and observed as early as E15. Western blot analysis of the 544aa-PAK3a and 559aa-PAK3b proves that these isoforms are equally expressed in the adult mouse brain. However, there is a significantly lower expression of the two 565aa-PAK3c and 580aa-PAK3cb. These alternative exons change drastically the biochemical properties of PAK3. For example: the PAK3b AID cannot inhibit PAK3a (Rousseau et al., 2003). Furthermore, PAK3b, c and cb display high constitutive kinase activity, and thus do not require GTPase activation. The difference in regulatory mechanisms and expression levels between PAK3a, PAK3b, PAK3c, and PAK3cb should be taken into account when estimating mutation pathogenicity. For example, one *PAK3* mutation was identified in the alternatively spliced exon b (Figure 3B) in a patient reported in a cohort of cerebral palsy (McMichael et al., 2015), even though new mining from a polymorphism database suggests that this mutation actually corresponds to a neutral polymorphism.

### Variations in functional domains

The PBD directly interacts with active GTP-loaded RAC1 and CDC42 (Lei et al., 2000). At first glance, the classical mechanism of activation by GTPases appears to be similar for the three PAKs, considering the similarity of the protein structures involved. The CDC42 and RAC1 interactive binding domain (CRIB) is a consensus sequence found in downstream effectors of RAC1 and CDC42 that constitutes the core of the PBD and is necessary for PAK activation and its subcellular location. Other PBD residues are also heavily involved in PAK selective interactions with GTPases (Knaus et al., 1998; Sun et al., 2019). Only one mutation affects this domain: the missense *PAK3* p.(Arg67Cys) mutation that is located at the limit of the PBD. PAK3 normally displays a higher affinity for CDC42 than for RAC1 according to *in vitro* assays, but the ID-associated p.(Arg67Cys) variant has more affinity for RAC1 than for CDC42, which changes its dynamics of activation and explains its property to induce immature dendritic spines in hippocampal cells (Kreis et al., 2007). GTPases of the Rac/Cdc42 family, that also interact with PAKs and may be involved in the pathophysiology of PAK variants, are still insufficiently explored in the context of *PAK*-associated disorders. Especially when taking into consideration that pathogenic variations in *RAC3* were recently identified in patients with developmental delay, brain anomalies, and facial dysmorphic features (Scala et al., 2022). Like *RAC1*, *RAC3* is ubiquitously expressed in the brain and evidence suggests that *RAC1* and *RAC3* share functions in neuron survival (Katayama et al., 2023). In double *Rac1/3*-KO mice, which display severe neuronal loss in the postnatal cerebral cortex, PAK is downregulated and the over-expression of constitutively active PAK1 rescues neuron survival and differentiation. All other GTPases of the Rac/Cdc42 family interact with PAKs but with no indication of their involvement in NDDs, as currently indicated by Decipher or ClinVar databases.

**FIGURE 4:**
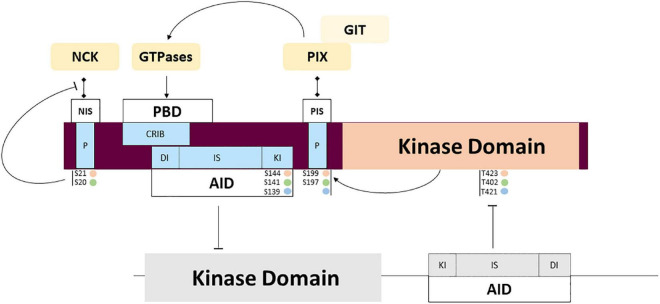
PAK regulation through partner interactions and intramolecular signaling. Complex mechanisms of regulation are triggered by interaction with activators and adaptors that may regulate activity and subcellular location. Several auto phosphorylation sites also participate in the regulation of interaction with the partners mentioned above, and the transitory location and activation of PAKs. Phosphorylation sites corresponding to PAKl (orange), PAK2 (green) and PAK3 (blue).

The AID is made of three highly conserved subdomains: a dimerization interface (DI) that contributes to the stabilization of PAK dimers, the inhibitory switch (IS) that partially overlaps the GTPase binding region, and a kinase inhibitor domain (KI) carboxy-terminal to the IS. PAK1 crystallographic structure highlights its ability to form dimers (Lei et al., 2000; Parrini et al., 2002). However, PAK3 forms preferentially heterodimers with PAK1 rather than homodimers, *in vitro* and *in vivo* (Combeau et al., 2012). Dimers are maintained inactive via the DI and the IS (Figure 4). The IS is folded against the catalytic region which favors the positioning of the KI domain into the catalytic cleft. The KI domain of each PAK within the dimer interacts with the catalytic site of the other PAK, thus inducing a *trans-inhibition* (Lei et al., 2000; Parrini et al., 2002). In their dimeric form, the CRIB domain in the PBD of each PAK is exposed at the surface, allowing PAK binding to an active small G protein.

*PAK1* mutations are mainly located in the regulatory domain (5/7). It is expected that mutations affecting the AID impair inhibitory interactions and induce constitutive activation (Tu and Wigler, 1999). To date, five *PAK1* mutations affecting the Ser110, Pro121, Tyr131 and Ser133 residues were thus identified in the AID and associated with ASD, ID and macrocephaly (Harms et al., 2018; Horn et al., 2019; Kernohan et al., 2019). Indirect experimental data strongly suggest that these PAK1 variants display constitutive or higher basal kinase activity (Harms et al., 2018). Moreover, the inhibitory domain of a given isoform acts on all three paralogs, and heterodimer formation facilitates crosstalk between PAK signaling. Consequently, mutations in the AID domain should lead to kinase activation of the variant and possibly heterodimerization defects, thus altering the kinase activity of other isoforms (Combeau et al., 2012).

In contrast, a greater proportion of missense mutations are located in the kinase domain of PAK2 (3/3) and *PAK3* (11/14). Several *PAK* mutations are located in the kinase domain: eleven are inactivating mutations and four are activating. Indeed, mutations in the kinase domain, such as the ones mostly found in *PAK2* and *PAK3*, usually disrupt kinase activity. This was particularly well described in the biochemical characterization of several *PAK3* mutations *in vitro* (Allen et al., 1998; Kreis et al., 2007; Magini et al., 2014; Duarte et al., 2020). *PAK2* mutations, also located in the kinase domain, are probably associated with loss of function. Structural data, computational analysis, and mutation compilations already established in the field of cancer may help analyzing the effect that kinase variants have on kinase activity, but biochemical characterization is often necessary (Dixit and Verkhivker, 2014).

Proline-rich sequences are involved in regulated interactions with important partners. Among the other conserved features, several highly conserved proline-rich regions found in the amino-terminal tail serve as binding sites for SH3 domains. Two sites are involved in binding adaptor proteins NCK1/2 and GRB2 which is involved in axonal guidance and synaptic transmission through PAK recruitment at the membrane. Isoform particularity is that the autophosphorylation of the PAK1 Serine 21 residue negatively regulates PAK/NCK interactions (Zhao et al., 2000). Interestingly, PAK3 was shown to preferentially bind NCK2/GRB4 compared to NCK1 and this interaction is not regulated by autophosphorylation (Thévenot et al., 2011). The absence of mutations identified in the NCK-binding domain is probably due to the current low sampling of mutations. The second proline-rich sequence is not completely conserved between the different PAK proteins and GRB2 interaction was only demonstrated for PAK1 (Puto et al., 2003). This pathway is probably impacted by mutation of the non-conventional kinesin KIF26A that inhibits GRB2, as observed in patients with severe brain malformations (Qian et al., 2022).

The central proline-rich sequence located between the AID and the kinase domain corresponds to an atypical SH3 binding domain that strongly binds the PAK interacting exchange factor (PIX/COOL) (Bagrodia et al., 1998; Manser et al., 1998). There are two PIX isoforms in mammals: the ubiquitously expressed ɑPIX, encoded by the *ARHGEF6* gene (Manser et al., 1998) and βPIX encoded by *ARHGEF7* and more specific to the brain (Koh et al., 2001). The numerous splice variants of PIX form different transient complexes with PAKs. These PIX-PAK complexes are regulated by phosphorylation. PIXs are also important in the recruitment of PAKs to the membrane and their activation. This complex located at the membrane enables the activation of GTPases and selectively favors PAK activation. The particularities of the different PAK isoforms in these molecular mechanisms are not known (Zhang, 2005; Zhou et al., 2016). ɑ/βPIX and GIT1/2 form larger complexes that interact with numerous proteins such as Paxillin and Shank, to coordinate their activity in cells and at synapses (Hashimoto et al., 2001; Parker et al., 2013; López-Colomé et al., 2017; Zhu et al., 2020). Disruption of the *ARHGEF6* gene leads to severe ID, dysmorphic features and sensorineural hearing loss (Kutsche et al., 2000). The mutation located near the PIX-binding sequence in *PAK3* was recently described in a girl with ID, microcephaly and immunological disease but without the biochemical analysis of the variant (Almutairi et al., 2021). The other proline-rich domains identified are not yet associated with partners and functions: indeed, PAK1 contains five proline-rich domains, while PAK2 has two and PAK3 has four. Whether all these proline-rich sequences bind the same partners with all three PAK isoforms is uncertain and has yet to be experimentally demonstrated.

The differences in peptide sequences also influence phosphosites. At the membrane, Rho-GTPase interactions induce a conformational change via the first event of *trans-phosphorylation* (threonine 423 for PAK1, 402 for PAK2 and 421 for PAK3). This restores some activity at the catalytic domain, which triggers *cis-phosphorylation* of sites, thus reinforcing kinase activity and completing the dissociation of dimers as well as maintaining monomers in an active conformation (Lei et al., 2000; Parrini et al., 2002; Pirruccello et al., 2006). No mutation affecting phosphosites has been described so far. Whether some *PAK* mutations can alter autophosphorylation or phosphorylation via other regulatory kinases has yet to be demonstrated. The caspase cleavage site, specific to PAK2, plays an important role in signaling but is not yet associated with NDDs (Jakobi et al., 2003).

PAK interactions with lipids, such as membrane lipids like PIP2, also participate in the opening of the kinase and its activation (Parrini et al., 2009; Malecka et al., 2013). PAKs are also enriched in dendritic spines (Zhang, 2005; Hayashi et al., 2007). As early as 2004, it was shown that PAKs copurify with synaptic markers (Hayashi et al., 2004). This analysis highlighted the colocalization of activated PAK with phospho-Cofilin and PSD95 upon neuronal activation in cortical neuron cultures and in organotypic hippocampal slices (Hayashi et al., 2004; Chen et al., 2007). Biochemical fractionation followed by Western blot analysis revealed the presence of active PAKs in the post-synaptic density (PSD) fractions of mouse brains, whereas inactive PAKs copurified mainly with presynaptic markers such as synaptophysin (Hayashi et al., 2004). However, fractionation experiments using adult mouse cortex suggest that PAK1 strongly localizes in the presynaptic fraction while PAK3 (especially PAK3a and PAK3b) co-purifies more with post-synaptic densities (Combeau et al., 2012). This suggests they may play different roles depending on their location and association with distinct synaptic elements.

## *PAK* expression patterns and mutation pathogenicity

**FIGURE 5:**
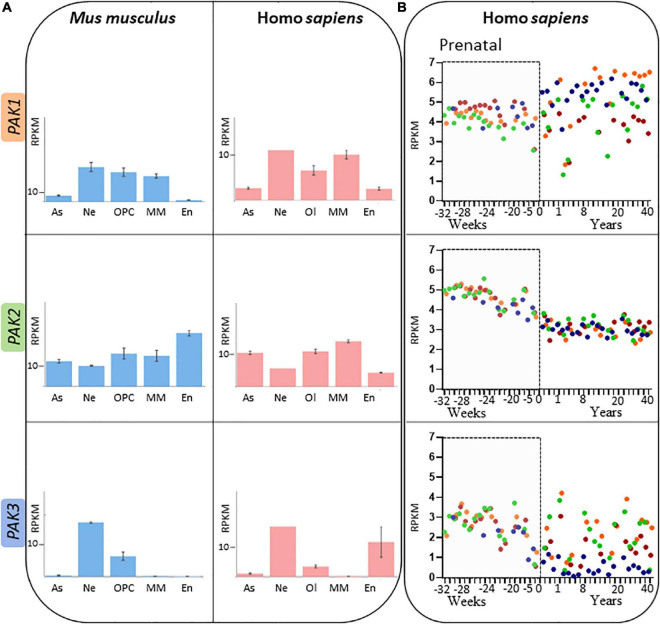
Spatio-temporal expression patterns of *PAK* isoforms **(A)** *PAK* expression depending on brain cell types, expressed in RPKM (reads per kilobase of exon per million reads mapped) in adult mouse (blue) and human (pink). Astrocytes (As), neurons (Ne), Oligodendrocyte precursor cells (OPC) or Oligodendrocyte (Ol), microglia/macrophages (MM) and endothelial cells (En). Data mining from brainrnaseq.org. **(B)** *PAK* expression throughout human lifespan in neurons of the cerebellar cortex (blue), hippocampus (green), striatum (brown), and frontal cortex (orange). Data mining from brainspan.org.

Group I *PAK*s share many structural and regulatory similarities, but their temporal and spatial expressions diverge. Globally, *PAK1* expression is high in muscles, in the spleen, and the brain except in the dentate gyrus (BrainSpan, 2022 *Atlas of the Developing Human Brain*) while *PAK2* expression is ubiquitous (Manser et al., 1995; Teo et al., 1995; Whale et al., 2011). *PAK3* is mostly expressed in the brain, with a particularly strong expression in the hippocampus. At the cellular level in the brain, *PAK1* is found only in neural lineage cells, *PAK2* is expressed in every cell types and *PAK3* can be found in neurons as well as oligodendrocyte progenitor cells (Figure 5A; Zhang et al., 2014; Maglorius Renkilaraj et al., 2017; Magne et al., 2021; Wang and Guo, 2022). However, some differences were noticed in *PAK* expression between mouse and human brain (Figure 5A; Civiero and Greggio, 2018; Zhang K. et al., 2022). Such differences should be taken into account when experiments are conducted on animal models and for extrapolation to humans.

*PAK* expression also differs along human lifespan (Figure 5B; BrainSpan, 2022: *Atlas of the Developing Human Brain*; Miller et al., 2014). The differences in pattern of expression may indicate that the different *PAKs* do not have the same role over the course of human brain development. *PAK* expression also presents variations depending on the brain region, which might reflect region-dependent specificities in addition to their concomitant functions. *PAK1* expression increases after birth in several regions such as the cerebellar and frontal cortices, but remains at a stable level in the striatum. *PAK2* expression profile appears to be similar in all brain regions (Figure 5B). Steadily decreasing after birth (Iossifov et al., 2014; Wang et al., 2018), *PAK3* expression is consistent throughout human brain development in the hippocampus, striatum and the frontal cortex but decreases after birth in the cerebellar cortex.

*PAK* expression is altered in several NDDs, suggesting that the decrease in PAK may be partly involved in cognitive or developmental symptoms. Therefore, the post-mortem meta-analysis of differentially expressed genes in the brain of ASD patients shows that neural *PAK1* expression is reduced in this pathology (Rahman et al., 2020). Hearing loss is correlated with ASD in children and this symptom may be due to the dysregulation of *PAK1* expression. Indeed, *PAK1* is highly expressed in the postnatal mouse cochlea, but the study of the *Pak1*-knockout mouse model showed that PAK1 deficiency downregulates cofilin phosphorylation and the expression of βII-spectrin, decreases the hair synapse density in the cochlea, and finally leads to hair cell apoptosis and severe hearing loss (Cheng et al., 2021).

Another example is *PAK3* downregulation in a model displaying iron deficiency-dependent cognitive alterations (Schachtschneider et al., 2016). *PAK3* is also one of the differentially expressed genes in BP and its expression is also regulated by valproate, a histone deacetylase inhibitor used to treat BP (Sinha et al., 2021). *PAK1* and *PAK3* mRNA levels are significantly reduced in the post-mortem brain of subjects affected with depression (Fuchsova et al., 2016). Transcriptomic analysis and genome-wide association in schizophrenia and bipolar disorder identify *PAK1* and *PAK3* as the differentially expressed genes along with several other genes in the RAC1/CDC42 pathway, highlighting the role of this pathway in these psychiatric disorders (Zhao et al., 2015).

Thus, it is important to consider *PAK* regulation of expression and the way it might change in the context of NDDs. Transcriptional and post-transcriptional regulations of each *PAK* present differences that may also change the way mutations affect brain development and functions. *PAK1* expression is activated via FOXO and E2F2 transcription factors and is inhibited by Rb (de la Torre-Ubieta et al., 2010; Sosa-García et al., 2015). *PAK2* transcriptional regulation is still an understudied subject, however, *PAK2* was found as differentially methylated in children with ASD (Jasoliya et al., 2022). *PAK3* expression is inhibited by Notch, ZEB1, SP8/9 and DLX1/2 transcription factors, highlighting its role in the proneural pathway (Souopgui, 2002; Cobos et al., 2007; Liu et al., 2019; Shen et al., 2019; Wei et al., 2019). *PAK3* transcription is also activated via Neurogenin and cJUN/AP-1 (Parker et al., 2013; Piccand et al., 2014). Several microRNAs (miRNAs) were identified in the regulation of *PAK* expression. miRNAs are gene regulators frequently involved in neurological disorders (Kim and Pak, 2020; Yao et al., 2020). At least seven *PAK1*-targeting miRNAs regulate *PAK1* transcription: miR-7, 34b, 96, 140-5p, 145, 485-5p, and 494 (Yue et al., 2016; Yao et al., 2020; Zhao et al., 2022). More than 19 microRNAs target directly *PAK2* expression, such as miR4779 (Koo and Kwon, 2018), miR-7-5p (Li et al., 2019) and miR455-3p (Hu et al., 2019). Several miRNA such as miR-134-5p, miR-1252, miR-125b-5p, miR193b-3p, miRNA-495, miRNA542 and miRNA133 were shown to be involved in *PAK3* downregulation, mainly in cancer (Chiurazzi and Pirozzi, 2016; Zhang et al., 2020; Tan et al., 2021). Interestingly, expression of the miR-495 is also involved in schizophrenia (Santarelli et al., 2019). Alternative polyadenylation (APA) are post-transcriptional events that regulate gene expression and that play a key role in cell proliferation and differentiation, and in neuronal functions. APA events may produce different protein isoforms. *PAK3* exhibits bimodality of distal APA usage in a type of GABAergic interneurons, the chandelier cells. These interneurons have specific spatial and temporal origins, target the axon initial segment of pyramidal neurons of the hippocampus and are implicated in brain disorders, including schizophrenia, epilepsy, and ASD (Yang et al., 2022).

To our knowledge, no data exists concerning the role of PAKs in other brain cells, except for the oligodendrocyte lineage (Maglorius Renkilaraj et al., 2017). Interestingly, MRI analysis of white matter reveals hypomyelination in patients bearing *PAK1* pathogenic variations whereas partial agenesis of corpus callosum and thin fiber tracts have been observed in some patients with *PAK3* mutations (6/16). *In vitro* experiments demonstrated that *PAK3* deletion impairs the differentiation of oligodendrocyte precursors, supporting a developmental and cell-autonomous role for *PAK3* during the first steps of myelination. These fragmentary data suggest that defects induced by *PAK* variations in oligodendrocytes or oligodendrocyte precursors may also account for some phenotypical traits observed in affected patients.

To summarize, these different PAK isoforms may be involved in one of the several crucial cellular mechanism that shape the brain, through proliferation and apoptosis, cell type specification, migration, arborization, and synaptic genesis. Nonetheless, experimental evidence attributing unique and specific functions to one isoform is currently lacking, making it difficult to link spatiotemporal expression characteristics to clinical phenotypes.

## The specific roles of PAKs isoforms

Among group I PAK isoforms, PAK1 is the most studied and is considered as representative of group I PAKs in general. Thus, there are currently more than sixty identified substrates of PAK1. These are involved in different major cellular processes such as proliferation, survival, motility and regulation of the actin cytoskeleton (Kanumuri et al., 2020; Liu et al., 2021). Such exhaustive list of specific substrates remains partial and cannot currently be compiled for the other isoforms (Ye and Field, 2012; Kumar and Li, 2016). Among the PAK-activated pathways, mentions should be made of the MAP kinase ERK pathways, the alteration of which leads to RASopathies, the Akt-PI3Kinase-mTor and Wnt pathways involved in ASD, the NF-κB pathway involved in apoptosis, among others (Yao et al., 2020; Caracci et al., 2021; Sharma and Mehan, 2021; Tartaglia et al., 2022). Many functions of the three PAK isoforms probably derive from their role in the regulation of actin cytoskeleton dynamics. It remains to be seen whether some of these functions are supported by only one of the three isoforms, which has not always been demonstrated experimentally. We can cite, for example, the following data. *PAK1* is involved in the proliferation and migration of neural progenitors in young mice (Pan et al., 2015) and dendritic maturation in the hippocampus (Dagliyan et al., 2017), which is essential to the regulation of postnatal cortical development. The abnormal increase in actin polymerization described in a mouse model of functional demyelination was caused by an over activation of PAK1 (Hu et al., 2016). *PAK2* probably plays an essential role during the first developmental stages as it regulates neuronal migration in the fetal brain and is, as such, essential for brain development. Interestingly, among *PAKs*, only *PAK2* suppression leads to the death of mouse embryos (Meng et al., 2005; Asrar et al., 2009; Wang et al., 2018). *PAK3* does not play the same role in cell cycle and cell division as *PAK1* and *PAK2.* While *PAK1* and *PAK2* have been characterized as oncogenes, *PAK3* is instead a probable tumor suppressor (Magne et al., 2021). In *xenopus laevis*, *PAK3* induces cell cycle arrest of precursors and their differentiation into neuroblasts during primitive neurogenesis (Souopgui, 2002). Furthermore, *PAK3* is also involved in axonal and dendritic arborization of immature interneurons and favors their tangential migration toward the cortex in mice (Cobos et al., 2007; Dai et al., 2014).

However, several studies have deciphered and attributed isoform-specific functions independent of actin. For example, PAK1 takes on actin-independent functions such as positive regulation of tonic GABA transmission via the downregulation of tonic endocannabinoïd (eCBs) secretion (Xia et al., 2018). The phosphorylation of CtBP1 by PAK1 is also a key step in synaptic vesicle retrieval in cultured hippocampal slices (Ivanova et al., 2020). Indeed, recordings of synaptic currents from CA1 neurons in *Pak1*-KO mice showed a decreased GABA signaling. This was also the case with acute PAK1 inhibition in hippocampal slices. Interestingly, the maturation of GABA function plays a major role in postnatal brain development (Deidda et al., 2014; Peerboom and Wierenga, 2021). PAK2 displays actin-independent pro-apoptotic functions, since PAK2, but not PAK1 nor PAK3, is cleaved by caspase 3, 8 and 10 in response to Fas signaling, which leads to PAK2-dependent apoptosis (Rudel and Bokoch, 1997; Fischer et al., 2006). PAK2 activated by RAC and CDC42 promotes cell survival while the caspase/PAK2 pathway is indeed pro-apoptotic (Marlin et al., 2011). Finally, one of PAK3 specific functions is the controls of AMPA receptor (AMPAR) trafficking via GluA1 subunit phosphorylation, upregulating the number of AMPAR at the membrane (Hussain et al., 2015).

Several data illustrate the link between PAK signaling and NDDs. PAKs were shown to regulate several proteins involved in NDDs, such as LIMK and cofilin (Figure 1). PAKs family members also interact with other downstream proteins that are dysregulated in NDDs or whose variations are causative factors in NDDs. For example, it was demonstrated that PAK1 binding to ERK2 facilitates ERK2 signaling, or activates their upstream activators RAF1 and MEK, which are involved in RASopathies (Sundberg-Smith et al., 2005; Motta et al., 2020; Liu et al., 2021). PAK1 is also required for the activation of NFκB pathway (Frost et al., 2000) via its modulation of the PPARy/NFκB signaling (Dammann et al., 2015). The expression of NFκB pro-inflammatory transcription factor is dysregulated in the brain of ASD patients and animal models of ASD. This dysregulation could be a pathogenic mechanism of neuro-inflammation involved in ASD (Young et al., 2011; Honarmand Tamizkar et al., 2021). Furthermore, PAK1, but not PAK2 nor PAK3, interacts with FRX family proteins involved in Fragile X syndrome, notably by phosphorylating FRX1 at Ser-420 (Say et al., 2010; Majumder et al., 2020). PAK2 modulates Wnt and Hedgehog signaling (Sementino et al., 2022). These proteins are essential to brain development and are involved in ASD and brain structural anomalies (Mulligan and Cheyette, 2017; Memi et al., 2018). Variations in the GluA1 subunit of AMPA receptors, one of PAK3-specific targets, are associated with moderate to severe ID and sometimes epilepsy, ASD, ADHD and movement disorders (Ismail et al., 2022).

## PAK dysregulation in neurodegenerative diseases: Cause or consequence?

Group I *PAK* dysfunctions and dysregulations, that are the root causes in several NDDs, are also involved in neurodegenerative diseases (Hayashi et al., 2004; Kreis et al., 2008; Ma et al., 2012). Numerous observations describe dysfunctions and abnormal decreases in the expression or activity of PAK during the evolution of neurodegenerative diseases such as Alzheimer’s, Parkinson’s and Huntington’s diseases, as well as some ataxias and prion diseases. These dysfunctions are often concomitant with dendritic spine abnormalities, emphasizing the question of whether PAK alteration is a cause or a consequence of synaptic deterioration. We can summarize the data as follows. In post-mortem brains of Alzheimer’s disease (AD) patients, PAK proteins are markedly reduced and their phosphorylated forms present abnormal localization, which is also correlated with abnormal cofilin activation and aggregates in neurites (Ma et al., 2008; Arsenault et al., 2013; Bories et al., 2017; Lauterborn et al., 2020). Concordant results were observed in the Tg2576 mouse model of AD which display peptide Aβ-containing plaques, but not apoptosis or microfibrillary tangles (Zhao et al., 2006). Interestingly, PAK inhibition in the brains of older mice triggers neuronal damage and cognitive deficits similar to those of the Tg2576 mice, clearly indicating a role of PAK in the Aβ pathology. It was shown in primary neuron cultures that interaction of PAK3 dominant-negative variant with the amyloid precursor protein (APP) blocks APP-mediated neuronal apoptosis in familial AD (McPhie et al., 2003). The C-terminal peptide cleaved from the APP at Asp664 may be responsible for these abnormal PAK activation (Nguyen et al., 2008). The inhibition of cofilin dephosphorylation in the Familial Autosomal dominant (FAD) mouse model of AD that express several pathogenic mutations in APP and Presinilin1 genes, decreases Aβ plaques, cellular defects and improves cognitive function, confirming the link between Aβ pathologies and actin cytoskeleton (Deng et al., 2016).

PAK1-mediated oligomerization of Huntingtin (HTT), which is independent of PAK1 kinase activity, is associated with Huntington’s disease (Luo et al., 2008). Interestingly, αPIX also enhances HTT aggregation via PAK1 (Eriguchi et al., 2010). Furthermore, HTT negatively regulates PAK2 apoptotic functions by preventing PAK2 cleavage via caspases: this protective function is retained by mutated *HTT*. These studies suggest that *PAK1* and *PAK2* have completely different associations with Huntington disease, PAK1 increases the toxicity of mutated *HTT* while PAK2 possibly has protective effects (Luo and Rubinsztein, 2009). PAK1 also regulates the expression of Ataxin-1 that is involved in spinocerebellar ataxia type I, suggesting that PAK1 inhibition may be a therapeutic approach in this orphan neurodegenerative disease (Bondar et al., 2018).

Synaptic activity requires a significant energy supply, provided mainly by the mitochondria. The routing of presynaptic mitochondria is under the control of a recently identified signaling pathway, involving AMP-activated protein kinase, which is mediated by PAKs via their kinase activity (Kong et al., 2016). This pathway regulates the recruitment of mitochondria and their anchoring to the vicinity of presynaptic terminals via myosin VI phosphorylation (Li et al., 2020). Defects in energy metabolism are also features of neurodegenerative diseases and could involve this AMPK/PAK/MYOVI pathway. It is interesting to note that the hippocampus, a key brain region for memory formation, is one of the cerebral structures most sensitive to hypoxia. However, the involvement of PAKs in this respect has not been documented (Schmidt-Kastner, 2015). PAK1 and PAK3 are also down-expressed in the post-mortem brains of Parkinson patients. In conclusion, alterations in PAK expression and activity are also involved in neurodegenerative pathologies, associated with excessive apoptosis, energy deficit, and dendritic spine shrinkage.

In summary, these numerous observations describe PAK down-expression, dysfunction, mislocation, and dysregulation throughout the progression of neurodegenerative diseases. PAKs are at the center of three signaling pathways involved in neurodegeneration, namely the regulation of dendritic spine dynamics, apoptosis and neuronal energy homeostasis. However, signaling pathways that link together synaptic defects, energy balance dysregulations, and abnormal neuronal death are complex and the role of PAK defects in these diseases remains unclear. A seminal experiment in mice showed that PAK inhibition causes cofilin anomalies and memory impairments similar to AD defects, strongly suggesting a causal role of PAK dysfunctions in AD-associated cognitive deficits (Zhao et al., 2006). Are PAK dysfunctions a cause in the emergence of neurodegenerative diseases in the patients with NDDs? At the moment, the absence of published prospective clinical follow up of patients with *PAK*-associated conditions prevents us from knowing the incidence of neurogenerative diseases on these patients. This has not been addressed by experimental approaches either. While the role of PAKs is well documented in worsening actin-dependent synaptic dysfunctions in AD, their role in apoptosis and energy failure during neurodegenerative disease progression is still elusive. Moreover, although several isoform specificities have been observed, the role of each PAK in these pathologies is still unclear. Nevertheless, recent data suggest that the activation of the acetylcholine muscarinic receptor improves aversive memory in striatal and accumbens nuclei via a Protein kinase C/Rac1/PAK pathway and may thus be a new therapeutic approach, at least for this type of behavioral symptoms (Yamahashi et al., 2022).

## Experiments in PAKs studies: Finding the right model

These recent and scarce data open the way toward a new understanding in the etiology of many cerebral pathologies. Yet, there is still much to uncover in the field of *PAK*-associated disorders. A deeper exploration of each *PAK* specificity, a more thorough investigation of the molecular mechanisms underlying *PAK* mutation pathogenicity and adapted therapeutic strategies are needed to improve diagnosis and treatment in *PAK*-related disorders. After the discovery of *PAK*s in 1994 (Manser et al., 1995) and after establishing their role in the regulation of cytoskeleton dynamics (Sells et al., 1997, 1999), studies conducted in cell and neuronal cultures helped characterizing PAKs regulation (Manser et al., 1998; Banerjee et al., 2002; Brown et al., 2002) and functions in migration (Causeret et al., 2009), neurite outgrowth (Daniels et al., 1998), spine morphogenesis, synapse formation and plasticity (Boda et al., 2004; Hayashi et al., 2007; Kreis et al., 2008; Dubos et al., 2012). Other studies have been conducted in animal models, widely used to understand mechanistic aspects of neurodevelopmental pathogenesis. Such models can be of translational significance or bring insight into fundamental mechanisms involved in NDDs (Fallah and Eubanks, 2020; Mariano et al., 2020; Rotaru et al., 2020). Even though some *PAK* studies have been conducted in non-mammalian models (Santiago-Medina et al., 2013; Magini et al., 2014), most are carried out in mouse models. Thus, *Pak1*-knockout mice have been generated and display MAPK signaling defects (Arias-Romero and Chernoff, 2008) and, accordingly, present slight immune deficiencies (Allen et al., 2009). LTP deficits and a decrease in NMDA activity-induced cofilin phosphorylation have also been identified in *Pak1*-KO mice (Asrar et al., 2009). The fact that the phenotype of *Pak1* and *Pak3* KO mice are relatively mild, while the double *Pak1/3* KO generates substantial cognitive loss, brain morphological abnormalities, as well as functional and structural synaptic defects suggests that these two isoforms have redundant functions allowing functional complementation (Meng et al., 2005; Asrar et al., 2009; Huang et al., 2011). Nevertheless, all *PAK1* variations generate GOF that cannot be compensated by other isoforms.

*Pak2*-knockout mice are not viable (Arias-Romero and Chernoff, 2008). However, *Pak2*^+/–^ mice have been studied and display ASD-related behaviors and reduced spine density, defective LTP and impaired actin polymerization (Wang et al., 2018), which is concordant with the effects of *Pak2* haploinsufficiency in humans. The high PAK2 expression during embryonic development, the lethality of its gene invalidation and its role in the establishment of cerebral vascularization, are in agreement with the fact that bi-allelic loss have not been identified in patients. However, it is surprising that no variant with gain of function has not been identified yet.

The deficits in late-LTP and CREB activity in the brain of *Pak3*-KO mice indicate a more important role of PAK3 in the actin-independent regulation of plasticity-associated genes (Meng et al., 2005). Interestingly, double *Pak1/Pak3*-knockout mice display hyperactivity, anxiety, learning and memory deficits, secondary microcephaly, reduced synaptic density but increased size of individual synapses and increased basal synaptic transmission, disrupted LTP and LTD, decreased cofilin regulation and reduced CREB activity (Huang et al., 2011). PAK1 displays a higher expression during development than in adulthood, in contrast to PAK3, suggesting that PAK3 has more developmental functions.

Thus, the global inhibition of PAKs in post-mitotic neurons of mice forebrain via the expression of the PAK kinase activity-inhibiting AID (*dnPak*) cause an increase in dendritic spine size, enhanced synaptic transmission and LTP as well as LTD deficits in the cortex (Hayashi et al., 2004). Consequently, mice expressing *dnPAK* displayed memory deficits. One can also wonder whether *Pak3*-AID only blocks kinase-activity-dependent functions, leaving PAK signaling at least partially functional. The AID of PAK3 was also used to post-natally block PAK activity in regions associated with social functions, which impaired social memory retrieval (Leung et al., 2018; Zhou and Jia, 2021). These models shed light on the roles of PAKs and how PAK disruption might affect brain functions. However, they only represent the loss of PAK proteins (in the case of *Pak*-knockouts) or loss of PAK kinase function (in the case of PAK inhibition via dn*Pak*) while *PAK1* mutations are usually gain of functions. There are also differences in *PAK3* nonsense mutations, that might lead to a complete loss of protein, and *PAK3* missense mutations that would have more variable effects.

New models, such as the knock-in mouse line which expresses the *Pak3-*Arg67Cys mutation, responsible for a moderate to severe ID in humans are expected to mimic more closely the precise biochemical alterations caused by PAK mutations. Thus the effects of a PAK3 variant displaying functional kinase activity, but with changes in partner interactions *in vitro*, has been studied and furthers our understanding pathogenic mechanisms underlying *PAK3* pathogenicity *in vivo*. The PAK3-Arg67Cys variant impacts newly generated neurons circuit integration in the adult mouse hippocampus, thus disturbing complex memory tasks performance such as pattern separation (Castillon et al., 2020). Through the study of animal models bearing *Pak* mutations, directly linking behavioral symptoms and brain dysfunctions to molecular mechanisms is now achievable. However, as shown in Figure 5, gene expression might not be the same between animal models and humans. Thus, patient-derived cell models are important to complement animal studies. PAK1 variants have been proposed to display high kinase activity in patient fibroblasts (Harms et al., 2018). A similar study was conducted in keratinocytes, which have a higher level of *PAK3* expression than fibroblasts (Magini et al., 2014). Obviously, the development of iPSCs from patient fibroblasts and models of embryonic bodies would help put together a more elaborated analysis of *PAK* mutation-dependent cell abnormalities. For example, the first data obtained for Down syndrome, the most frequent cause of ID, confirms the hyperactivation of the DsCam/PAK1 module in patient-derived iPSCs and highlight the possibility to target PAKs in this disease (Tang et al., 2021).

## PAKs as therapeutic targets in brain diseases

Researchers are confronted with the contradiction of wanting, on one hand, to target a molecule in a strategy applicable to many psychiatric and neurodevelopmental disorders, and on the other hand, to develop approaches applicable to specific cases, with the perspective of personalized medicine. Cofilin which is a focal point of the Rho GTPase pathway, is a therapeutic target that has already been validated to correct behavioral and synaptic plasticity defects in several NDD models (Shaw and Bamburg, 2017). PAKs are also points of convergence and therefore potential therapeutic targets. Indeed, since the phenotypes associated with some NDDs appear to be due to exacerbations of the Rac/Cdc42/PAK pathway activity, PAK inhibition may be an interesting therapeutic approach. Several PAK inhibitors were developed and display high affinity and selectivity against group I-PAKs (Semenova and Chernoff, 2017). First evidence of this concept is that the expression of an autoinhibitory domain of PAK3, which targets all three group I PAKs improves the cellular and behavioral phenotype of Fmr1 KO mice, a model of Fragile X syndrome (Hayashi et al., 2007). Interestingly the bioavailable and brain–permeant catalytic inhibitor of group I PAK, FRAX486, rescues the spine defects, seizures, stereotypical behavior, and hyperactivity displayed by *Frm1*-KO mice (Dolan et al., 2013). FRAX486 also rescues neurobehavioral alterations in a mouse model of CDKL5 disorders, probably via the regulation of the complex between the main CDKL5 substrate ARHGEF2 and PAK1 (Zenke et al., 2004; Fuchs et al., 2022). PAK over-activation was also observed in neurons cultured from *Disc1*-KO mice, a mouse model of schizophrenia. DISC1 downregulates Kalirin7, a potent RAC1-activating GEF, upon NMDAR activation (Hayashi-Takagi et al., 2014). PAK inhibition using FRAX486 prevents DISC1 RNAi-induced spine deterioration (prophylactic effect) and reverses already existing spine deterioration triggered by DISC1 RNAi (treatment effect) in neuronal cultures. It also partially recues spine morphology in adult mice (Hayashi-Takagi et al., 2014). In the case of neurofibromatosis type 1 (NF1) there is also cognitive and learning disabilities that are associated to MAP Kinase activation and dendritic spine regulation. PAK1 gene invalidation or pharmacological inhibition of PAK by intracerebral injection of the IPA3 inhibitor restores social interaction and memory deficits (Molosh et al., 2014). PAK1 activity is increased in a mouse model of hereditary demyelinating neuropathy of demyelination, in which disease progression is associated with an increase of actin polymerization at the nerve myelin junction (Hu et al., 2016). Pharmacological inhibition of PAK using PF3758309 normalized levels of F-actin, completely prevented the progression of the myelin junction disruption, and restored nerve conduction.

Several PAK inhibitors target the ATP binding site of the catalytic domain via a competitive mechanism (FRAX compounds, PF3758309), but the high identity of peptide sequences within the catalytic domain prevents poor PAK-isoform specificity of inhibitors toward the three PAK isoforms. Another inhibitor, the IPA-3 compound, is a brain-permeant allosteric inhibitor that binds to the PDB/AID, impairs GTPase binding, and thus prevents group I-PAK activation but has no effect on already activated kinases (Deacon et al., 2008). However, recently, a compound (NVS-PAK1-1) that limits the PAK access to ATP was characterized as the first PAK1 specific inhibitor, especially compared to PAK2 (Semenova and Chernoff, 2017). It would be interesting to test to what extent these PAK inhibitors could constitute therapeutic approaches in cases where mutations confer kinase activity over-activation (6/23). However, for the majority of variants (15/23), mutations in the *PAK* genes lead to the loss of kinase activity (Figure 3C). In these situations, it would probably be more appropriate to target a downstream molecule, such as cofilin.

## Conclusion and perspectives

**FIGURE 6:**
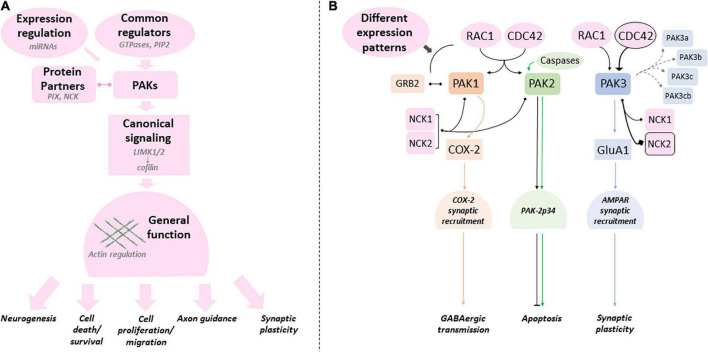
Canonical and specific PAK-dependent processes **(A)** Group I PAKs share several regulatory mechanisms, protein partners and substrates (canonical processes in pink - examples in light gray). *PAK* mutations can affect any of these processes. **(B)** Several studies emphasize the differences between PAKs (canonical processes in pink can still present quantitative differences). PAKl (orange), PAK2 (green) and PAK3 (blue) were shown to have their own specificities (colors matching the corresponding PAK). Protein partners and regulators that are favored by PAK are emphasized with a black frame. Mutations can have different effects on these specific processes and explain the variations found between PAKs and intragenic variability.

*PAK* mutations are rare events but their genetic traceability as monogenic defects, their total penetrance, the expanding collection of human mutations and curated phenotypes, make the study of these genes ideal to discover the biological mechanisms that go awry in common human NDDs. The exploration of fundamental and novel biological processes can greatly benefit from the study of rare genetic disorders (Lee et al., 2020). Group I PAKs occupy a central place in neuron physiology by being RAC1/CDC42 downstream effectors as well as the convergence point of many other pathways that control cell fate, motility, cell proliferation and apoptosis. Furthermore, an increasing number of experiments also indicate that PAKs are heavily involved in synaptic transmission and plasticity. Nevertheless, clinical data and fundamental experiments in controlled paradigms strongly suggest that these kinases present both redundant and isoform-specific functions essential for the development and processes of the brain and other organs (Figure 6).

In summary, *PAK1* mutations are dominant gain of function, *PAK2* mutations generate haploinsufficiency, whereas *PAK3* pathogenic variations have various functional consequences. The genotype/phenotype correlation is relatively easy to comprehend for the first two genes, associated with developmental delay and macrocephaly or with ASD, respectively. However, this relation remains to be established for PAK3. The main goal is to identify the molecular and cellular processes at the foundation of the differences between each isoform genotype/phenotype correlation. Why *PAK1* mutations are GOF and *PAK2* mutations are LOF is elusive, and there is still a gap preventing the full comprehension of the link between isoform-specific functions and the phenotypes associated with mutations in each *PAK* gene. More specifically, the biochemical alterations caused by different *PAK* mutations are still understudied. It is necessary to obtain a deeper understanding of the expression, localization, regulation, substrates and partners of each *PAK* isoform in the more global context of PAK interactome in order to gauge the impact of mutations on these processes. As listed above, several publications already demonstrate the role of these kinases in several neurological disorders such as Fragile X, neurofibromatosis, Down syndrome and several rare diseases caused by synaptic gene mutations, but also in neurodegenerative diseases such as Alzheimer’s, Huntington’s, Parkinson’s and some ataxias. With very few exceptions, it does not currently seem possible to decipher whether the three PAKs underlie the same dysfunctions in some or all of these neurological diseases. Understanding this is a challenge for future translational studies. It is perhaps also interesting to note that PAKs are associated with COVID pathologies, in particular *PAK1* in the virus infection process and *PAK3* in certain immunological signatures of patients (Yu et al., 2021; Zhang K. et al., 2022). This could be linked to the neurological damage associated with the long forms of COVID-19 (Monje and Iwasaki, 2022). Many experimental approaches already brought a proof of the concept that group I *PAK*s are interesting therapeutic targets in NDDs and neuropsychiatric disorders. The joint effort in the fields of cancerology, neurodegenerative disorders, rare and neurodevelopmental diseases will greatly advance our knowledge on *PAK* functions, dysfunctions and adapted therapeutic strategies in the view of personalized treatments. One of the remaining weaknesses in the study of the genotype/phenotype relationship in *PAK*-associated diseases is the rarity of reported cases. However, the efforts of clinicians and geneticists to implement clinical databases provide access to more genetic and clinical data, thus allowing the genotype/phenotype correlation to be established more precisely. Clinician and researcher collaborations, improved experimental models, precise biochemical characterization of variants and conceptual frameworks in the context of convergent neurosciences could be the missing stone to fully comprehend the biological mechanisms underlying mutation pathogenicity and to successfully translate research findings into clinical practices (Willsey et al., 2017; Srivastava et al., 2018).

## Author contributions

MD and J-VB conceptualized the review and wrote it. AV and AT supervised the clinical data. SP-G and VR followed the successive steps of writing with their advice and corrections. All authors contributed to the article and approved the submitted version.
